# Clinicians’ Perceptions towards Precision Medicine Tools for Cardiovascular Disease Risk Stratification in South Africa

**DOI:** 10.3390/jpm12091360

**Published:** 2022-08-24

**Authors:** Michelle Kamp, Oliver Pain, Andrew May, Cathryn M. Lewis, Michèle Ramsay

**Affiliations:** 1 Division of Human Genetics, National Health Laboratory Service and School of Pathology, Faculty of Health Sciences, The University of the Witwatersrand, Johannesburg 2001, South Africa; 2 Sydney Brenner Institute for Molecular Bioscience, Faculty of Health Sciences, University of the Witwatersrand, Johannesburg 2193, South Africa; 3 Maurice Wohl Clinical Neuroscience Institute, Department of Basic and Clinical Neuroscience, Institute of Psychiatry, Psychology and Neuroscience, King’s College London, London SE5 9RT, UK; 4 Social, Genetic and Developmental Psychiatry Centre, Institute of Psychiatry, Psychology & Neuroscience, King’s College London, London SE5 8AF, UK; 5 Department of Medical and Molecular Genetics, Faculty of Life Sciences and Medicine, King’s College London, London SE1 9RT, UK

**Keywords:** precision medicine, polygenic risk scores, clinician attitudes, clinical utility, clinical implementation, cardiovascular disease

## Abstract

Cardiovascular diseases (CVDs) are a leading cause of mortality and morbidity in South Africa. Risk stratification is the preferred approach to disease prevention, but identifying patients at high risk for CVD remains challenging. Assessing genetic risk could improve stratification and inform a clinically relevant precision medicine (PM) approach. Clinicians are critical to PM adoption, thus, this study explores practicing clinicians’ perceptions of PM-based CVD risk stratification in South Africa’s public health setting. Practicing clinicians (*n* = 109) at four teaching hospitals in Johannesburg, South Africa, completed an electronic self-administered survey. The effect of demographic and professional characteristics on PM-based CVD risk stratification perceptions was assessed. Fewer than 25% of respondents used clinical genetic testing, and 14% had formal genetics training. 78% had a low mean knowledge score, with higher scores associated with genetic training (*p* < 0.0005) and research involvement (*p* < 0.05). Despite limited knowledge and resources, 84% perceived PM approaches positively. 57% felt confident in applying the PM-based approach, with those already undertaking CVD risk stratification more confident (*p* < 0.001). High cost and limited access to genetics services are key barriers. Integrating genetic information into established clinical tools will likely increase confidence in using PM approaches. Addressing the genetics training gap and investment into the country’s genomics capacity is needed to advance PM in South Africa.

## 1. Introduction

Cardiovascular diseases (CVDs) are the leading cause of mortality globally [1]. South Africans are at risk as they have a high and increasing prevalence of obesity, hypertension and undiagnosed diabetes [2]. In 2019, CVDs were the leading cause of mortality (15.85% of deaths) and the third leading cause of morbidity (7.08% of DALYs) in South Africa [3]. This growing epidemic is exacerbated by the lack of preventative screening, early diagnosis, and inadequate access to healthcare services. The burden associated with CVDs emphasizes the need for intensified efforts to identify high-risk patients and improve prevention, diagnosis and management.

Globally, and within South Africa, a risk-based prevention strategy is the most widely accepted approach to disease prevention, with high-risk individuals offered preventative medication and encouraged to adopt healthier lifestyles [4,5]. Clinical practice guidelines advocate risk calculators to estimate the 10-year risk of disease in order to identify and target high-risk individuals [5,6,7,8]. Risk stratification is calculated from multiple demographic and clinical factors, such as age, sex, smoking status and blood lipid levels. Despite widespread use, it is estimated that these calculators fail to identify up to 40% of high-risk patients [9,10], an underestimation likely greater in Africa, where scores are yet to be validated in local populations [11]. Moreover, although genetics is a known risk factor for CVD [12,13], current clinical tools do not assess genetic risk directly.

The summation of genetic variants into a polygenic risk score (PRS), a single value estimate of an individual’s total genetic risk burden for disease, is able to stratify individuals according to their genetic risk [13,14]. Integrating PRSs with traditional risk factors can improve the prediction of CVD and associated traits [10,15,16,17]. Such integrated tools, or precision medicine (PM) approaches, where individual genetic variation, lifestyle, and health history are leveraged to tailor prevention and treatment strategies, have the potential to improve patient care and reduce the burden of CVDs on healthcare systems [18,19,20].

Despite the anticipated benefits of PM, significant barriers to integrating genomics into clinical practice remain [18,19,20,21,22]. In addition to the scientific, educational, ethical, legal, and social barriers, the type of testing and physician-based characteristics may also play a role [23]. Given that clinicians are the interface between healthcare systems and patients, understanding their perceptions and willingness to utilize PM-based approaches is critical to ensuring patients reap the benefits of PM in a timely and cost-effective manner, especially where healthcare systems are fragile and under-resourced [23,24].

Little is known about healthcare providers’ perceptions of PM tools in Africa [25,26]. Most research has explored medical professionals’ views in higher and middle-income countries with robust healthcare settings [23,27,28,29,30,31,32,33,34,35]. This study aimed to address this gap by exploring medical doctors’ perceptions of a PM-based tool for CVD risk prediction in South Africa. We report our assessment of the findings and discuss clinicians’ needs and the broader barriers that need to be addressed to introduce PM-based approaches in the South African public health setting.

## 2. Materials and Methods

### 2.1. Study Design and Participants

Study participants were drawn from clinicians primarily affiliated with four public academic hospitals in Johannesburg, South Africa (Charlotte Maxeke Johannesburg Academic Hospital, Chris Hani Baragwanath Academic Hospital, Helen Joseph Academic Hospital, and Rahima Moosa Mother and Child Hospital). Four hospitals were selected to ensure an adequately sized and representative sample. Clinicians at all levels (intern, medical officer, registrar, and specialist) across all medical specializations were eligible for inclusion. Clinicians were excluded if they had not completed their medical training and/or were not affiliated with the selected hospitals.

The study was approved by the University of the Witwatersrand Human Research Ethics Committee (Medical), Johannesburg, South Africa (Approval #M210355).

### 2.2. Study Questionnaire

To assess clinician experiences and attitudes towards PM-based tools for CVD risk stratification, a 107-item electronic survey was developed. The survey was based on similar validated surveys [33,34,35], with adaptations to the local context, and was developed in consultation with a multidisciplinary group of academics and clinicians. Four specialists, two clinicians, and two geneticists assessed the survey face validity and ensured question clarity. The survey instrument was tested amongst five clinicians for content, design, and readability, and revised accordingly based on feedback.

The survey was organized into eight sections (Table 1 and Table S1) and administered in identical order (i.e., no section/item randomization). Items consisted of close-ended questions formatted using a five-point Likert-type rating scale. Items were scored in ascending order for all participants, with higher scores on rating scales implying increased self-perceived knowledge, perception, and confidence of PM-based stratification tools. A higher rating indicated a greater degree of benefit or concern associated with the specific item in the case of benefits and concerns, and an increased level of impact indicated that the barrier would be a greater hindrance to tool implementation. *‘I prefer not to answer’* was available for all questions, and free text options were available for respondents wishing to provide additional clarification.

Clinicians working in South Africa have diverse educational backgrounds, with education and training from multiple institutions within South Africa and abroad. To ensure that all respondents had a similar baseline understanding of PM and related concepts, a compulsory 15-min educational video was included in the questionnaire (available on request). To determine whether the video had successfully relayed the pertinent topics, Section 3 included nine true or false questions. A score of 78% (7 out of 9) and above was interpreted as successfully understanding the topics.

### 2.3. Data Collection and Analyses

Invitation to participate was extended via email to clinicians over a six-month period, from 10 September 2021 to 31 March 2022. Consenting respondents completed the survey, which was hosted online (REDCap). Due to low initial uptake, researchers requested permission to attend departmental clinical academic meetings, where the study aim and survey could be better outlined prior to inviting participation. Of the 15 departments approached, nine accepted (Internal Medicine, Cardiology, Clinical Genetics, Clinical Pathology, Emergency Medicine, Pediatrics and Child Health, and Surgery). Study data were collected and managed using REDCap (Research Electronic Data Capture) (version 12.5.4, Vanderbilt University, Nashville, TN, USA) tools hosted at the University of the Witwatersrand [36,37].

Self-perceived knowledge, perception and confidence (Sections 3, 4 and 6; Table 1) were calculated as continuous scores by converting responses to a numerical scale and summing the participants’ responses, e.g., Knowledge: level of understanding scale converted to numeric scale (No understanding = 0, Little understanding = 1, Some understanding = 2, moderate understanding = 3, Expert understanding = 4) and the total score calculated by summing scores for each question (maximum score = 24). Mean knowledge score (MKS) was derived by dividing the participants’ total score by 6 (number of questions). The mean perception (MPS) and confidence (MCS) scores were calculated similarly, with a maximum obtainable score of 4. However, MPS was calculated over five items. Regarding the nine items for assessing comprehension following the educational video, one point was given if the correct answer was chosen, and a score of zero was given if the wrong answer or *‘I do not know’* was chosen.

For descriptive comparisons, participants were dichotomized into high/low categories for the MKS and MCS scores using a breakpoint mean score of 2 (with low being ≤ 2, high > 2). Similarly, respondents were dichotomized into negative/positive categories for MPS scores at a breakpoint mean score of 2. As categorizing a continuous variable causes a loss of appreciable information, inferential analysis was restricted to mean scores. Similarly, age and number of years of clinical experience were dichotomized by the median.

Differences in MKS, MPS, and MCS distributions between groups were assessed with parametric (*t*-test and ANOVA) or non-parametric tests (Mann–Whitney or Kruskal–Wallis), where appropriate, followed by multivariate regression analysis (see Table 2 for study variables). Practice level and medical specialty were removed from the model due to collinearity with other predictors (Figure S1). All analyses were done using RStudio [38] (version 1.1.456), the psych (version 1.8.4; [39]) and ggplot2 (version 3.0.0; [40]) packages. Study responses were presented as percentages (95% confidence intervals; CI) and means (standard deviation; SD). Statistical significance was accepted at a *p*-value < 0.05.

## 3. Results

### 3.1. Participant Characteristics and Exposure to Genetics and Cardiovascular Disease Screening

A total of 114 participants completed the survey. Five respondents were excluded as the respondents were non-patient-facing, resulting in a final sample of 109 participants. An accurate response rate could not be calculated, as the exact number of clinicians who received the questionnaire is unknown. Power calculations using Raosoft^®^ showed a sample size of at least one hundred was required for an 8% margin of error and 95% confidence interval, assuming a response distribution of 50%. Table 3 presents respondent characteristics and exposure to study-related clinical variables. One-quarter of respondents were interns, designated ‘trainees’ in subsequent analyses, with a maximum of two years clinical experience and no medical specialization. Over three-quarters were consultant clinicians. 25% of these practiced within internal medicine disciplines, such as Cardiology and Endocrinology, and 70% within non-internal medicine disciplines, including Pediatrics and Clinical Genetics. The mean (SD) age of the study population was 37.1 years (12.5), with a mean of 11.8 years (12.1) of clinical experience. Age and clinical experience were highly correlated (r = 0.97, SE = 0.03). Consequently, inferential analysis was restricted to clinical experience as to avoid multicollinearity.

Two-thirds of respondents undertake CVD risk stratification in their clinical practice. Sixty percent of those use a stratification tool to determine risk, with the Framingham Coronary Heart Disease Risk Score being the most used (80%). 58% undertake CVD screening in response to risk factors, including family history and abnormal laboratory results. Clinicians are confident in the tool, with the majority sharing results with patients (86%) and using them to guide treatment (96%). Most of those who do not undertake screening (68%) indicate that screening is not standard practice in their patient population, e.g., pediatrics and clinical genetics.

Overall exposure to genetics and PM was low. Fifteen percent of respondents indicated they had formal genetics training, and approximately one quarter had performed genetic testing in their clinical practice, although testing is infrequent (Table 3)

### 3.2. Genetics and Precision Medicine Knowledge

**Figure 1:**
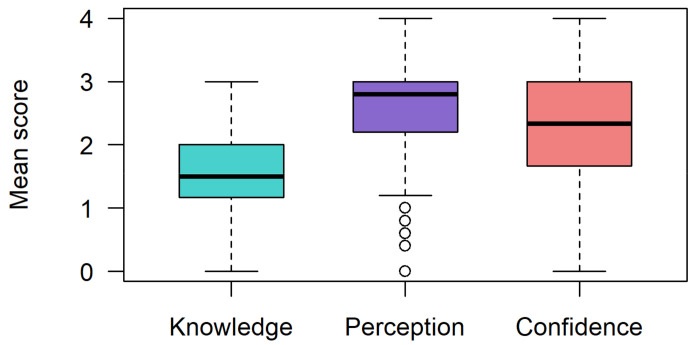
Respondents’ overall mean knowledge, perception and confidence scores. Mean scores calculated per individual across items relating to knowledge, perception and confidence.

Participants rated their level of understanding of six genetics and PM-related topics. The mean knowledge scores per individual across all six items were low (mean (SD) = 1.57 (0.64); max score = 4) (Figure 1) resulting in 80% (*n* = 108; 95% CI 70.5–86.5) of respondents categorized as having ‘low knowledge’. A single individual indicated they had ‘extensive knowledge’ across all six themes, and no respondents answered all six items as ‘expert’.

**Figure 2:**
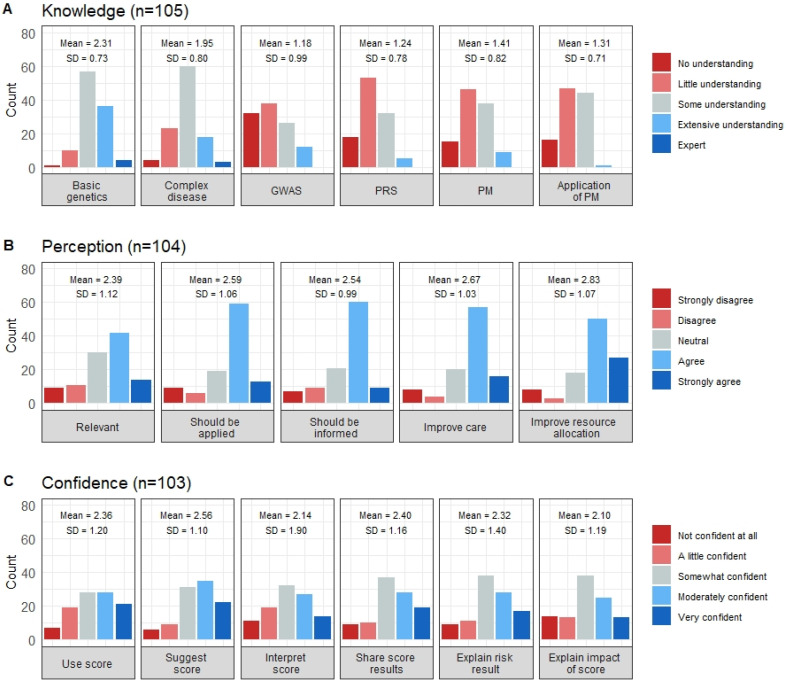
Respondents’ knowledge, perception and confidence of items relating to PM-based CVD risk stratification. The distribution of the clinicians’ responses regarding (**A**) Genetics and PM knowledge, (**B**) Perceptions towards PM-based CVD risk stratification and (**C**) Confidence in applying PM-based CVD risk stratification in clinical practice. GWAS—Genome-wide association studies, PRS—Polygenic risk scores, PM—Precision Medicine.

Variability in understanding shifted towards little or no knowledge for newer genetic concepts, with approximately two-thirds of individuals indicating little or no understanding of Genome-Wide Association Study (GWAS) and PRS (Figure 2A). Regarding the assessment of knowledge following the introductory video, 97% of respondents obtained a score of 78% or higher, suggesting the educational lecture successfully relayed the study’s pertinent points and assisted in providing context to survey questions.

### 3.3. Perceptions toward Precision Medicine-Based CVD Risk Stratification

Overall, clinicians had a positive perception of a PM-based tool for CVD risk stratification (mean (SD) = 2.60 (0.90); max score = 4). Three-quarters of respondents had a positive mean perception score when categorized as positive or negative (*n* = 104; 76%, 95% CI 67.0–83.7) (Figure 1). Despite just over half of respondents believing such a tool would be relevant in their practice, at least two-thirds believed it should be applied in clinical practice (69%) and that it would improve care and prevention of CVD (70%) and national resource allocation (73%) (Figure 2B).

### 3.4. Confidence in Applying a Precision Medicine-Based CVD Risk Stratification Tool in Their Practice Settings

Confidence (self-perceived) scores showed considerable variation (mean (SD) = 2.31 (1.04); max score = 4). Approximately half of respondents (55%) considered themselves confident across all confidence items (Figure 1). Otherwise, confidence depended on the action required within the clinical pathway. Clinicians were most confident in suggesting and using the risk score, whereas 29% had little to no confidence in interpreting the risk score and explaining any potential adverse insurance policy impacts that may arise from risk information (27% little to no confidence) (Figure 2C). Forty percent of respondents indicated they would be moderately or very comfortable with adapting prevention and treatment strategies based on score results. However, a greater proportion of respondents were comfortable with adapting their treatment strategies (22%) than their prevention approaches (13%).

### 3.5. Factors Influencing Knowledge, Perceptions, and Confidence

We assessed how respondents’ characteristics influenced their knowledge, perceptions, and self-confidence toward PM-based CVD risk stratification (Table 4). Medical specialty, sex, and clinical experience did not significantly influence knowledge levels. Medical research involvement had a small effect on knowledge, with those involved in research having increased self-perceptions of knowledge [1.71 (0.61) vs. 1.38 (0.61); *p* < 0.01; effect size r = 0.26]. Previous genetics training had a large effect on knowledge, with those having had training having significantly higher mean knowledge scores [2.26 (0.62) vs. 1.42 (0.54); *p* < 5 × 10^−5^; effect size R = 0.76]. Similarly, medical research involvement influenced overall self-confidence in applying PM-based risk stratification [2.57 (0.96) vs. 2.01 (1.06); *p* < 0.05; effect size R = 0.26]. Self-confidence scores were also influenced, with a moderate effect, by whether respondents currently undertook CVD risk stratification in their clinical practice or not [2.65 (1.10) vs. 1.93 (0.96); *p* < 0.05; effect size η^2^= 0.07]. Conducting CVD screening on a regular or infrequent basis resulted in a significantly higher mean perception score, and with a large effect [Yes: 2.89 (0.63), Sometimes: 2.65 (0.88) vs. No: 2.11 (1.03); *p* < 0.0005; effect size η^2^= 0.13].

### 3.6. Multivariable Analysis of the Factors Affecting Knowledge, Perceptions, and Confidence

When adjusting for study predictors, medical research involvement and genetics training remained positively associated with mean knowledge score, with the model explaining 30% of the overall variance. Similarly, for confidence and perception, medical research involvement (confidence only) and CVD screening status remained associated. However, the influence of CVD screening increased, and the model explained 14% and 13%, respectively. The model revealed the medical group to be associated with the mean knowledge and mean confidence score when adjusting for other predictors (Table S2).

### 3.7. Benefits and Concerns of a PM-Based CVD Risk Stratification

**Figure 3:**
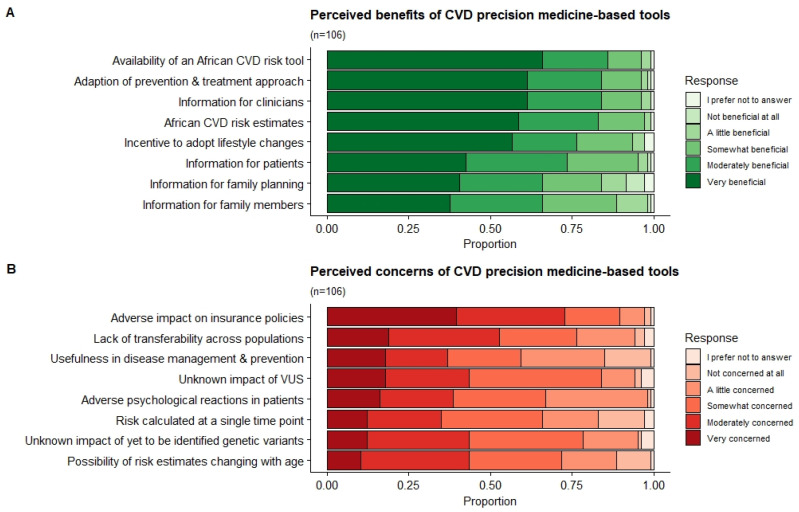
Perceived benefits and concerns of a PM-based CVD risk stratification. The distribution of the clinicians’ responses relating to the perceived benefits (**A**) and concerns (**B**) of a PM-based CVD risk stratification in the South African public setting.

Figure 3 presents the distribution of the clinicians’ responses regarding perceived benefits (A) and concerns (B) of a PM-based CVD risk stratification in the South African public setting. Respondents believed the main benefits associated with PM-based CVD risk stratification was the potential availability of a CVD risk score tailored to African populations (very beneficial and moderately beneficial *n* = 91; 86%; 95% CI: 77.4–91.6), followed by the adaption of prevention and treatment strategies (very beneficial and moderately beneficial (*n* = 89; 84%: 95% CI: 75.3–90.1). The most concerning issue for clinicians was the potential adverse impact that score results may have on a patient’s life and insurance policies (very concerned and moderately concerned *n* = 77; 73%; 95% CI: 63.0–80.6), and the lack of transferability of current PM risk that scores have across populations (very concerned and moderately concerned *n* = 56; 53%; 95% CI: 42.9–62.5).

### 3.8. Clinician’s Expectations: Time Horizon, Funding, and Expected Role

Assessing clinicians’ expectations of PM-based tools for CVD risk stratification in South Africa’s public revealed that clinicians anticipate a clinically relevant tool for African populations to be available in the longer term. Over one third (36%) expect the tool within the next six to ten years, and another third in more than ten years (39%). Half of the respondents feel that funding would be the responsibility of multiple players, but at least a third (35%) believe that national and provincial governments should be the primary funder. Despite the expected wait and funding complexities, 85% of respondents indicate that they are likely or very likely to alter their CVD screening approach if a tool becomes available (Table S3).

When asking clinicians what they believe their expected role will be in the implementation of PM-based CVD risk stratification, approximately two-thirds identify themselves as having the primary role in traditional patient-facing activities (referring the patient for testing (73%), sharing results with the patient (62%), and using results to guide prevention and treatment approaches (70%). In contrast, at least half of the respondents recognize that interpreting (55%) and explaining results to patients (54%) will require additional expertise and thus a multidisciplinary team (Table S3).

### 3.9. Perceived Barriers to the Implementation of PM-Based CVD Risk Stratification

**Figure 4:**
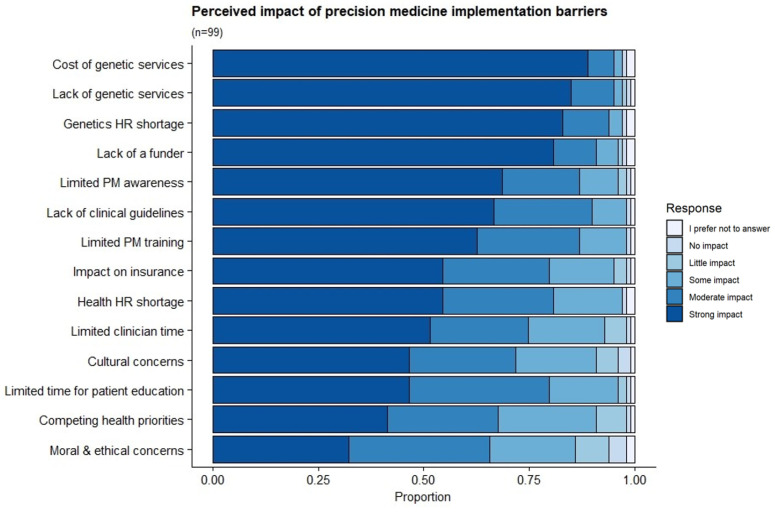
Perceived impact of PM-based CVD risk stratification implementation barriers in the South African public setting. The distribution of responses regarding the barriers facing the implementation of PM-based CVD risk stratification in the South African public setting.

According to respondents, the top implementation barriers facing PM-based CVD risk stratification in South Africa’s public health setting are the cost (strong impact = 89%), and the lack of genetics services (strong impact = 85%) and associated shortage in genetics personnel (strong impact = 83%). Other barriers included the lack of a funder (strong impact = 81%), the absence of clinical guidelines (strong impact = 66%) and limited training in PM and related concepts (62%) (Figure 4).

## 4. Discussion

As little is known about healthcare providers’ perceptions of PM tools in Africa, this study aimed to explore medical doctors’ perceptions of a PM-based tool for CVD risk prediction in South Africa. Results revealed clinical exposure to, and knowledge of genetics is limited in the South African public health setting, but most perceive PM approaches positively. Confidence in applying PM-based CVD risk stratification is variable, with those involved in research currently undertaking CVD risk stratification being more confident.

### 4.1. Positive Perceptions of PM-Based Tools despite Knowledge Gaps and Resource Constraints

Our study identified low rates of genomic testing, coupled with low self-perceived knowledge of genetics and PM-related concepts among clinicians and trainees practicing in South Africa’s public health setting. Despite limited understanding and resource challenges, over two-thirds of respondents expressed positive perceptions of PM-based stratification tools. Such findings are not dissimilar to existing literature, which reveals low self-perceived knowledge but positive perceptions amongst healthcare providers across medical specializations, geographies and economic regions [23,25,26,30,32,33,34,35,41,42,43,44,45]. Assessment across five Implementing GeNomics In pracTicE (IGNITE) sites in the USA involving differing precision medicine applications revealed most clinicians believed genetic testing to be clinically useful. However, only a third thought they were adequately trained to provide care for genetically “high-risk” patients [23].

Although the value of integrated CVD risk scores was recognized, the perceived relevance of PM-based risk stratification to individual practice was variable and linked to varying practice standards across medical specialties, highlighting the need for clinician inclusion in the development of appropriate PM tools. The availability of risk estimates tailored to African populations was considered to be of particular value to providers, but the current limited transferability of PM-based approaches across ancestries [46] was also a key concern.

### 4.2. Addressing the Genetics Education Gap Is Paramount to Successful Adoption

Given that exposure to genetic testing and PM approaches is likely to increase with decreasing costs and increasing efforts to understand African genetic variation, these findings support prior calls for increased training and educational resources [23,30,41,47,48]. Furthermore, the limited genetic literacy amongst South African patients further necessitates additional support from healthcare providers to benefit from genetics and PM.

Lower self-perceived knowledge amongst consultants compared to trainees suggests that exposure to PM and related concepts in undergraduate training may have shifted in recent years. Alternatively, inexperienced clinicians may not be aware of the complexities of testing in a clinical setting as yet. Although different, mean knowledge scores for both groups were low, and highlight training requirements at undergraduate and practice levels. Genetics curricula can go unnoticed by clinicians as it is often integrated into the content of a course [49]. Simply increasing the volume and duration of genetic curricula is unlikely to be helpful, as advances in genomics do not occur in isolation and compete with other compelling learning demands [47]. Specialized courses and qualifications, although increasingly available, require substantial commitments and have relatively low uptake amongst non-genetic specialists [49]. Genomics education incorporated into healthcare providers’ usual work activities, including departmental presentations and clinical meetings, has been identified as a preferred training strategy [50,51]. The most appropriate training approach is debated. However, there is consensus that education should address common misconceptions related to genetics, emphasize the clinical applications of genomics while not negating the complexity of the associated basic science, and ensure that clinicians can correctly utilize genetic-based risk estimations in their clinical practice [47,49,52].

Practicing clinicians have indicated a preference for online continued medical education, conferences, peer-reviewed literature, and in-clinic training [28,51,53]. Historically, most continued medical education has been passive and didactic, with limited impact on changing practice. The most impactful approaches are those that are interactive, use a variety of instructional techniques, and focus on outcomes considered important by healthcare professionals. The appropriateness of such activities in South Africa will need to be further investigated, prioritizing culturally sensitive approaches that consider language differences and delivery format challenges.

### 4.3. Integrated Models Compatible with Current Clinical Practices Are Needed

In this study, confidence in utilizing the approach was variable and was directly influenced by previous CVD risk stratification exposure. Literature suggests that clinicians lack confidence in using and implementing new genomic tests and precision medicine approaches, and that this low confidence is linked to limited knowledge of genetics and related topics [28,30,33,42,50,54]. However, increased confidence has been associated with increased exposure to testing, or when the application is clearly defined and given within the context of a specific disease [23,55]. For example, oncologists already using genetic testing in clinical practice reported the highest confidence in using multimarker tumor panel results to guide patient care [55]. Increased confidence amongst those already using CVD stratification algorithms suggests that successfully integrating genetic risk assessment into existing clinical frameworks requires strategies compatible with current practice.

The areas clinicians lacked the most confidence in were related to the interpretation of score results and explaining potential impacts on insurance policies, which may reflect the lack of understanding of the genetic components of the score and the legislative framework. Respondents indicated a strong preference for a multidisciplinary model approach to testing, particularly interpreting score results and explaining such to patients. Multidisciplinary teams require specialized genetics skillsets, such as clinical geneticists and genetic counselors, which may not be feasible in the context of a skills shortage in South Africa. Increasing clinicians’ comfort with using genomics in routine care may reduce the reliance on genetic specialists and improve the feasibility of genomic medicine over the long term. Additionally, the development of guidelines for PM-based scoring may help improve understanding in these areas and would support testing practices.

### 4.4. Funding Shortfalls and Skills Shortages Hinder PM Adoption in Resource-Scarce Settings

Similar to previous research and other settings, surveyed clinicians identified the affordability and accessibility of genetic services and genetics skills shortage as major impediments to PM implementation in South Africa’s public health settings [18,25,30,41,42,56]. These challenges are not unique to PM and successful innovative models in funding and delivering care, especially within limited resource settings, should be explored.

Additional barriers raised from studies in well-resourced settings include those related to organizational structures (management, organizational culture and/or decentralized care), lack of clinical guidelines and the poor coordination of tests relative to treatment decisions [33]. Nephrologists in Australia highlighted barriers relating to their specific practice setting, e.g., leadership endorsement and goal setting [42], and medical oncologists in Canada identified patient genomic literacy and the current clinical utility of genomics as key challenges [35]. Although not identified as primary barriers in this South African study, these challenges are likely to be critical in the South African public setting as system-level barriers are addressed. Stakeholders will need to consider these challenges when developing a PM implementation roadmap, and leverage lessons learned as resource challenges are overcome and healthcare systems stabilize.

This study was novel in that it included a compulsory educational video before responding to the questionnaire. The purpose was to provide context and some exposure to PM across the responder group. However, several limitations to the study design merit discussion. Over and above the limited sample size and potential responder biases associated with online convenience sampling strategies [57], the study makes use of a newly developed instrument which has not yet been extensively tested for reliability and validity. There may have been issues with biased response sets (e.g., “faking good”) that would be difficult to detect in the absence of reverse-scored items [58]. Reassuringly, however, our results are intuitive and align with literature trends, diminishing concerns around the psychometric rigor of the instrument.

Lastly, for convenience, the study survey was administered to clinical practitioners based in large, relatively well-funded urban medical centers. Consequently, respondents in this study are unlikely to be representative of health professionals across South Africa. Understanding primary care providers’ perceptions of and willingness to utilize PM-based tools in the primary care setting should be explored. Primary care provision is the foundation of care in South Africa, serving most communities, especially in rural areas, and where CVD risk stratification could have the greatest benefit [59,60,61]. Successful PM utilization requires multi-stakeholder co-operation, and thus the views of patients, insurers, policy making bodies and governments, should also be explored in follow up studies.

This study sheds light on an under-researched area of South African healthcare and contributes to a greater understanding of what will be required to successfully implement PM in South Africa, and potentially other resource-constrained environments.

## 5. Conclusions

Practicing clinicians have a positive perception of PM-based CVD risk stratification despite limited genomic knowledge, and they have a desire to adopt such tools in their practices. Limiting adoption barriers requires developing tools that leverage existing clinical approaches. The advancement of PM in South Africa requires an active effort to address the gap in genetic training whilst simultaneously investing in the country’s genomics capacity, including both skill force and infrastructure.

## Figures and Tables

**Table 1:** Survey organization and associated scales used.

Section	Theme	No. of Items	Scale
1	Demographic and professional	10	Not applicable
	information		
2	Exposure: CVD risk stratification, and	21	Not applicable
	genetics training and testing		
3	Knowledge: Genetics and PM, and	15	Understanding: *None*, *Little*, *Some*, *Moderate*, *Expert*
	educational video		Agreement: *True*, *False*, *I do not know*
4	Perception toward PM-based CVD risk stratification	5	Agreement: *Strongly disagree*, *Disagree*, *Neutral*, *Agree*, *Strongly agree*
5	Benefits and concerns of PM-based CVD risk stratification	19	Benefit/concern: *Not*, *A little*, *Somewhat*, *Moderately*, *Very*
6	Confidence in applying PM-based CVD risk stratification	8	Confidence: *None*, *A little*, *Somewhat*, *Moderately*, *Very*
7	Expectations in applying PM-based CVD risk stratification	9	Role: *None*, *Supporting*, *Primary*, *Not sure*
			Adoption likelihood: *Very unlikely*, *Unlikely*, *No difference*, *Likely*, *Very likely*
8	Barriers to implementing PM-based CVD risk stratification	15	Impact: *None*, *Little*, *Some*, *Moderate*, *Strong*

**Table 2:** Categorical and continuous predictor variables used for analysis in this study.

Variable	Description	Type (Range)		Categories
**Outcome variables**				
Mean knowledge score (MKS)	Average self-reported knowledge relating to genetics and PM	Continuous (0–4)		Not applicable
Mean perception score (MPS)	Average self-reported perception relating to value of PM-based CVD risk stratification tool	Continuous (0–4)		Not applicable
Mean confidence score (MCS)	Average self-reported confidence relating to implementation of PM-based CVD risk stratification tool	Continuous (0–4)		Not applicable
**Predictor variable**				
Sex	Reported sex	Categorical		Female
				Male
Medical group	Practitioner type based on self-reported practice level and years of experience	Categorical		Consultant
				Trainee
Clinical experience	Number of years conducting clinical duties	Categorical		<8 years
				≥8 years
Postgraduate qualifications	Have a postgraduate qualification in addition to a medical degree	Categorical	Yes	
			No	
Involvement in research	Are involved in medical research activities	Categorical	Yes	
			No	
Genetics or PM training	Have training specifically relating to genetics and/or PM	Categorical	Yes	
			No	
CVD stratification in clinical practice	Conducts CVD screening in their clinical practice	Categorical	Yes	
			Sometimes	
			No	

CVD—Cardiovascular disease, PM—Precision Medicine.

**Table 3:** Respondents’ demographics, professional characteristics and study-related clinical exposures (*n* = 109).

Variable	Detail	N (%)
Hospital affiliation	Chris Hani Baragwanath Academic Hospital	23 (21.1)
	Charlotte Maxeke Johannesburg Academic Hospital	36 (33.0)
	Helen Joseph Hospital	13 (11.9)
	Rahima Moosa Mother and Child Hospital	32 (29.4)
	I prefer not to answer	5 (4.6)
Medical specialty	Internal medicine	21 (19.3)
	Non-internal medicine	57 (52.3)
	Not yet specialized (trainee)	27 (24.8)
	I prefer not to answer	4 (3.7)
Sex	Female	67 (61.5)
	Male	42 (38.5)
Age	<33 years	57 (51.4)
	≥33 years	51 (46.8)
	I prefer not to answer	1 (0.9)
Medical group	Consultant	82 (75.2)
	Trainee	27 (24.8)
Clinical experience	<8 years	55 (50.5)
	≥8 years	50 (45.9)
Postgraduate qualifications	Yes	66 (60.6)
	No	41 (37.6)
	I prefer not to answer	2 (1.8)
Involvement in medical research	Yes	59 (54.1)
	No	50 (45.9)
CVD stratification in clinical practice	Yes	41 (37.6)
	Sometimes	31 (28.4)
	No	36 (33.0)
	I prefer not to answer	1 (0.9)
CVD stratification using a risk score	Yes	25 (34.7)
(*n* = 72)	Sometimes	19 (26.4)
	No	28 (38.9)
Genetics training	Yes	17 (15.6)
	No	91 (83.5)
	I prefer not to answer	1 (0.9)
Genetics testing in clinical practice	Yes	31 (28.4)
	No	78 (71.6)
Conditions genetics testing discussed * (*n* = 31)	Monogenic disorders	30 (96.8)
	Cancers	16 (51.6)
	CVD	6 (19.4)
	Nutrigenomics	1 (3.2)
	Pharmacogenomics	2 (6.5)
Genetic testing frequency (*n* = 31)	1 patient every 2 to 3 months	13 (41.9)
	1 patient per month	2 (6.5)
	2 to 5 patients per month	6 (19.4)
	6 to 10 patients per month	2 (6.5)
	11 to 20 patients per month	3 (9.7)
	20+ patients per month	3 (9.7)
	I prefer not to answer	2 (6.5)

* Each respondent could select multiple options.

**Table 4:** Predictors’ influence on respondents’ mean (SD) knowledge, confidence, and perception scores. *p* value is assessing differences in mean scores across variable subgroups.

Variable	Mean (SD) Knowledge Score (*n* = 107)	*p* Value	Mean (SD) Confidence Score (*n* = 104)	*p* Value	Mean (SD) Perception Score (*n* = 106)	*p* Value
**Medical specialty**						
Internal medicine	1.67 (0.67)		2.70 (0.94)		2.95 (0.64)	*0.052*
Non-internal medicine	1.47 (0.62)	*0.310*	2.12 (1.09)	*0.098*	2.40 (0.98)	
Not yet specialized	1.66 (0.62)		2.30 (0.92)		2.58 (0.85)	
**Sex**						
Female	1.59 (0.67)	*0.682*	2.41 (0.98)	*0.404*	2.45 (1.11)	*0.632*
Male	1.54 (0.61)		2.26 (1.08)		2.64 (0.74)	
**Medical group**						
Clinician	1.52 (0.64)	*0.318*	2.26 (1.08)	*0.933*	2.54 (0.93)	0.969
Trainee	1.66 (0.62)		2.30 (0.92)		2.58 (0.85)	
**Practice level**						
Specialist	1.61 (0.66)	*0.470*	2.25 (1.27)	*0.897*	2.48 (0.90)	*0.526*
Registrar	1.41 (0.65)		2.32 (0.81)		2.71 (1.05)	
Medical officer	1.48 (0.53)		2.56 (0.86)		2.60 (0.82)	
Intern	1.66 (0.62)		2.30 (0.92)		2.58 (0.85)	
**Clinical experience**						
<8 years	1.59 (0.60)	*0.580*	2.32 (0.84)	*0.651*	2.59 (0.86)	*0.945*
≥8 years	1.53 (0.67)		2.31 (1.23)		2.55 (0.95)	
**Postgrad. qualifications**						
Yes	1.62 (0.68)	*0.195*	2.32 (1.17)	*0.490*	2.62 (0.890)	*0.317*
No	1.45 (0.53)		2.27 (0.78)		2.48 (0.93)	
**Medical research involvement**						
Yes	1.71 (0.61)	0.007 **	2.57 (0.96)	0.007 **	2.69 (0.87)	*0.249*
No	1.38 (0.61)		2.01 (1.06)		2.43 (0.92)	
**Genetics training**						
Yes	2.26 (0.62)	3.518 × 10^−5^ ***	2.46 (0.86)	*0.720*	2.96 (0.61)	*0.146*
No	1.42 (0.54)		2.29 (1.17)		2.50 (0.93)	
**CVD screening**						
Yes	1.75 (0.59)	*0.074*	2.65 (1.10)	0.010 *	2.89 (0.63)	4.162 × 10^−4^ ***
No	1.46 (0.63)		1.93 (0.96)		2.11 (1.03)	
Sometimes	1.45 (0.65)		2.33 (0.94)		2.65 (0.88)	

* Postgrad.—Postgraduate, CVD—cardiovascular diseases; *p*-value thresholds: *** = *p* ≤ 0.001, ** = *p* ≤ 0.01, * = *p* ≤ 0.05.

## Data Availability

Available on reasonable request to the authors.

## Supplementary Materials

The following supporting information can be downloaded at: https://www.mdpi.com/article/10.3390/jpm12091360/s1, Figure S1: Correlation matrix of predictors: Mean knowledge score, mean perception score and mean confidence score; Table S1: Survey questions, Table S2: Multivariate analysis, Table S3: Clinicians’ expectations of PM-based risk stratification.
