# Supplementary material for: Clinicians’ Perceptions towards Precision Medicine Tools for Cardiovascular Disease Risk Stratification in South Africa
Source: J Pers Med. 2022 Aug 24;12(9):1360. doi: 10.3390/jpm12091360 (PMC9505828; doi:10.3390/jpm12091360)
Supplement: Supplementary file 1 [file jpm-12-01360-s001.zip › jpm-1784291-supplementary.pdf]

Supplementary data

Figure S1: Correlation matrix of study predictor variables for (A) Mean knowledge score (n=107), (B) Mean perception score (n=105), and (C) Mean confidence score (n=103)

A. Mean knowledge score with all study predictors

	MKS	Medical specialty	Sex	Medical group	Practice level	Clinical experience (years)	Postgraduate qualifications	Medical research involvement	Genetics training	CVD screening
MKS		0.05	0.06	0.1	-0.05	-0.08	0.16	0.23	0.49	0.16
Medical specialty	0.05		-0.11	0.83	-0.74	-0.59	-0.42	-0.51	0.04	-0.29
Sex	0.06	-0.11		-0.1	0.14	0.15	-0.02	0.02	0.08	-0.01
Medical group	0.1	0.83	-0.1		-0.84	0.57	-0.42	0.5	-0.01	-0.03
Practice level	-0.05	-0.74	0.14	-0.84		0.82	0.48	0.43	0.07	0
Clinical experience (years)	-0.08	-0.59	0.15	0.57	0.82		0.43	0.22	-0.07	0
Postgraduate qualifications	0.16	-0.42	-0.02	-0.42	0.48	0.43		0.37	0.03	0.05
Medical research involvement	0.23	-0.51	0.02	0.5	0.43	0.22	0.37		0.19	0
Genetics training	0.49	0.04	0.08	-0.01	0.07	-0.07	0.03	0.19		0.07
CVD screening	0.16	-0.29	-0.01	-0.03	0	0	0.05	0	0.07	

B. Mean perception score with all study predictors

	MPS	Medical specialty	Sex	Medical group	Practice level	Clinical experience (years)	Postgraduate qualifications	Medical research involvement	Genetics training	CVD screening
MPS		-0.13	-0.11	0	-0.02	-0.01	0.05	0.16	0.18	0.37
Medical specialty	-0.13		-0.11	0.83	-0.75	-0.59	-0.42	-0.52	0.04	-0.29
Sex	-0.11	-0.11		-0.1	0.14	0.14	-0.02	0.02	0.08	-0.01
Medical group	0	0.83	-0.1		-0.85	-0.58	-0.42	-0.51	-0.01	-0.03
Practice level	-0.02	-0.75	0.14	-0.85		0.82	0.49	0.43	0.07	0
Clinical experience (years)	-0.01	-0.59	0.14	-0.58	0.82		0.44	0.22	-0.08	0
Postgraduate qualifications	0.05	-0.42	-0.02	-0.42	0.49	0.44		0.38	0.04	0.05
Medical research involvement	0.16	-0.52	0.02	-0.51	0.43	0.22	0.38		0.19	0
Genetics training	0.18	0.04	0.08	-0.01	0.07	-0.08	0.04	0.19		0.07
CVD screening	0.37	-0.29	-0.01	-0.03	0	0	0.05	0	0.07	

C. Mean confidence score with all study predictors

	MCS	Medical specialty	Sex	Medical group	Practice level	Clinical experience (years)	Postgraduate qualifications	Medical research involvement	Genetics training	CVD screening
MCS		-0.11	0.08	0	-0.05	-0.02	0.04	0.26	0.06	0.27
Medical specialty	-0.11		-0.11	0.83	-0.75	-0.59	-0.43	-0.52	0.04	-0.3
Sex	0.08	-0.11		-0.09	0.13	0.12	0.02	0.04	0.09	0.02
Medical group	0	0.83	-0.09		-0.85	-0.57	-0.44	-0.53	-0.02	-0.04
Practice level	-0.05	-0.75	0.13	-0.85		0.82	0.51	0.45	0.08	0.01
Clinical experience (years)	-0.02	-0.59	0.12	-0.57	0.82		0.48	0.22	-0.08	0
Postgraduate qualifications	0.04	-0.43	0.02	-0.44	0.51	0.48		0.36	0.03	0.02
Medical research involvement	0.26	-0.52	0.04	-0.53	0.45	0.22	0.36		0.18	-0.03
Genetics training	0.06	0.04	0.09	-0.02	0.08	-0.08	0.03	0.18		0.06
CVD screening	0.27	-0.3	0.02	-0.04	0.01	0	0.02	-0.03	0.06	

Table S1: Survey questions

Clinicians' perceptions of incorporating a precision medicine-based cardiovascular disease risk stratification approach in South Africa

PARTICIPANT DEMOGRAPHICS AND OTHER CHARACTERISTICS

1. Please select your primary hospital affiliation:

(Chris Hani Baragwanath Academic Hospital / Charlotte Maxeke Johannesburg Academic Hospital / Helen Joseph Hospital / Rahima Moosa Mother and Child Hospital / I prefer not to answer)

2. What is your medical speciality?

(Internal Medicine / Obstetrics and Gynaecology / Orthopaedics / Paediatrics / Surgery / Other / I prefer not to answer)

3. Within Internal Medicine, what is your sub-speciality?

(Cardiology / Endocrinology / Hematology/Oncology / Infectious Diseases / Nephrology / Neurology / Pulmonology / Rheumatology / Gastroenterology / Dermatology /I prefer not to answer)

If other, please specify.

4. What is your current level of practice?

(Specialist / Registrar / Medical officer / Community service officer / Intern / Other / I prefer not to answer)

If other, please specify.

5. How many years of experience do you have in clinical practice (in years)?

(Start from the year after you graduated, including internship and community service, if you prefer not to answer, please put 000)

6. At which Institution did you study towards your medical degree?

(University of Cape Town / University of the Free State / University of KwaZulu-Natal / University of Limpopo / Sefako Makgatho Health Sciences University (previously known as MEDUNSA) / Stellenbosch University / University of Pretoria / University of the Witwatersrand / Walter Sisulu University / Other / I prefer not to answer)

If other, please specify.

7. Do you have any additional qualifications (postgraduate study) in addition to your medical degree?

(Yes / No / I prefer not to answer)

8. Are you actively involved in medical research?

(Yes / No / I prefer not to answer)

9. What is your sex?

(Female / Male / I prefer not to answer)

10. What is your age (in years)?

(If you prefer not to answer, please type 000)

CVD SCREENING EXPOSURE

1. Do you currently assess patient CVD risk in your clinical practice?

(Yes, No / Sometimes / I prefer not to answer)

2. When do you assess patient CVD risk?

(Initial consultation of all new patients / At every consultation / When patients present with risk factors / At the discretion of the attending physician / Other / I prefer not to answer)

If other, please specify when you assess CVD risk.

3. Do you use a CVD risk stratification tool to assess patient risk for CVD?
(Yes / No / Sometimes / I prefer not to answer)

4. Which tool do you most often use to assess CVD risk?
(Framingham risk score / Globorisk / Pooled Cohort Risk Equation (PCE) / QRISK / Systematic Coronary Risk Evaluation (SCORE) algorithm / WHO-CVD / Other / I prefer not to answer)

If other, please specify which tool you use to assess CVD risk

5. How confident do you feel in USING the CVD risk score assessment tool?
(Not confident at all / A little confident / Somewhat confident / Moderately confident / Very confident / I prefer not to answer)

6. How confident do you feel in INTERPRETING the results of the CVD risk score assessment tool?
(Not confident at all / A little confident / Somewhat confident / Moderately confident / Very confident / I prefer not to answer)

7. Do you share the results of the risk the CVD risk score assessment with the patient?
(Yes / No / Sometimes / I prefer not to answer)

8. Does the outcome of the risk score assessment guide?
(Yes / No / Sometimes / I prefer not to answer)

9. Why do you not assess patient CVD risk?
(Patients present with competing health issues / Patients referred from another doctor / Patients already considered high or very high risk for CVD / Insufficient time to assess CVD risk / Lack of funding for testing / Not standard practice / Other / I prefer not to answer

If other, please elaborate why you do not assess CVD risk.

GENETICS AND PRECISION MEDICINE EXPOSURE

1. Have you completed genetic or precision medicine related training or education?
(Yes / No / I prefer not to answer)

2. Please specify which training or education you have completed?
(A genetics module as part of my undergraduate medical training / A precision medicine module as part of my undergraduate medical training / A genetics short course after graduating / A precision medicine short course after graduating / A postgraduate genetics qualification / A postgraduate precision medicine qualification / Other / I prefer not to answer)
*(Selecting multiple options is possible)

If other, please specify.

3. Have you discussed genetic testing/precision-based medicine with a patient?
(Yes / No / Sometimes / I prefer not to answer)

4. For which diseases have you discussed genetic testing/precision-based medicine?
(Monogenic disorders (cystic fibrosis, familial hypercholesterolaemia, etc.) / Cancers (Breast, prostate, etc.) / Nutrigenomics / Pharmacogenomics / Cardiovascular diseases /Other / I prefer not to answer
*(Selecting multiple options is possible)

If other, please specify.

5. With how many patients do you discuss genetic testing/precision-based medicine with on a monthly basis?
   (Less than 1 patient per month / 1 patient every 2 to 3 months / 1 patient / 2 to 5 patients / 6 to 10 patients / 11 to 20 patients / More than 20 patients / I prefer not to answer)

6. Have you OFFERED a patient genetic testing/precision-based medicine to determine their disease risk?
(Yes / No / I prefer not to answer)

7. Have you ORDERED genetic/precision-medicine testing to determine patient disease risk?
(Yes / No / I prefer not to answer)

8. Have you REFERRED a patient for genetic/precision medicine testing to determine their disease risk?
(Yes / No / I prefer not to answer)

9. Have you RETURNED the results of genetic/precision medicine testing to a patient?
(Yes / No / I prefer not to answer)

10. Have you COUNSELLED patients on the results of their genetic/precision medicine testing?
(Yes / No / I prefer not to answer)

11. Have you APPLIED RESULTS from genetic tests to the treatment and management (drug choice, dosing, monitoring, etc.) of a patient?
(Yes / No / I prefer not to answer)

12. How regularly do you apply genetic testing results to the treatment and management of your patient?
(Never / Rarely / Sometimes / Often / Always I prefer not to answer)

GENETICS AND PRECISION MEDICINE KNOWLEDGE
Please select the corresponding box that BEST fits your level of understanding with each topic.

1. Basic genetic principles (Mendelian inheritance, penetrance, somatic vs. germline mutation, etc.).
(No understanding / Little understanding / Some understanding / Extensive understanding / Expert / I prefer not to answer)

2. Complex disease genetics (Coronary artery disease, diabetes, etc.)
(No understanding / Little understanding / Some understanding / Extensive understanding / Expert / I prefer not to answer)

3. Genome Wide Association Studies (GWAS)
(No understanding / Little understanding / Some understanding / Extensive understanding / Expert / I prefer not to answer)

4. Polygenic risk scores (PRS)
(No understanding / Little understanding / Some understanding / Extensive understanding / Expert / I prefer not to answer)

5. Precision medicine
(No understanding / Little understanding / Some understanding / Extensive understanding / Expert / I prefer not to answer)

6. When and how to incorporate precision medicine into practice
(No understanding / Little understanding / Some understanding / Extensive understanding / Expert / I prefer not to answer)

GENETICS AND PRECISION MEDICINE KNOWLEDGE
Please select the corresponding box that BEST fits your level of agreement with each statement.

1. A complex disease is caused by the interaction of multiple gene variants and environmental factors
(True / False / Do not know / I prefer not to answer)

2. Modifiable risk factors include smoking, alcohol intake and physical activity levels.
(True / False / Do not know / I prefer not to answer)

3. Examples of complex diseases include cancer and heart disease.
(True / False / Do not know / I prefer not to answer)

4. Risk stratification aims to stratify patient populations into high-, medium- and low-risk for improved care and better resource allocation.

(True / False / Do not know / I prefer not to answer)

5. Precision medicine aims to use an individual's genetic profile and other relevant clinical information to guide decisions regarding disease prevention, diagnosis, and treatment.

(True / False / Do not know / I prefer not to answer)

6. Precision medicine is a medical model that proposes a 'one-drug-fits-all' model.

(True / False / Do not know / I prefer not to answer)

7. Polygenic risk is a cumulative measure of genetic risk using multiple genetic variants

(True / False / Do not know / I prefer not to answer)

8. Polygenic risk scores are transferable across all ethnicities

(True / False / Do not know / I prefer not to answer)

9. Polygenic risk score results provide insight into a patient's total risk for disease.

(True / False / Do not know / I prefer not to answer)

PERCEPTIONS TOWARDS A PRECISION MEDICINE-BASED CVD RISK STRATIFICATION APPROACH IN SOUTH AFRICA

In relation to the precision medicine-based CVD risk stratification tool proposed in the video, please select the corresponding box that BEST fits your level of agreement with each statement:

1. Precision medicine-based CVD risk stratification is relevant to my current clinical practice.

(Strongly disagree / Disagree / Neutral / Agree / Strongly agree / I prefer not to answer)

2. Precision medicine-based CVD risk stratification should be applied to my clinical practice.

(Strongly disagree / Disagree / Neutral / Agree / Strongly agree / I prefer not to answer)

3. I should be able to provide information on the appropriate use of precision medicine-based CVD risk stratification.

(Strongly disagree / Disagree / Neutral / Agree / Strongly agree / I prefer not to answer)

4. Precision medicine-based CVD risk stratification will improve my ability to more effectively prevent and treat CVD.

(Strongly disagree / Disagree / Neutral / Agree / Strongly agree / I prefer not to answer)

5. Precision medicine-based CVD risk stratification will improve national resource allocation (expenditure, etc.) for CVD disease prevention and management.

(Strongly disagree / Disagree / Neutral / Agree / Strongly agree / I prefer not to answer)

CONCERNS

In relation to the precision medicine-based CVD risk stratification tool proposed, please select the corresponding box that BEST fits your level of concern with each statement:

1. The potential for adverse psychological reactions in patients receiving CVD risk information.

(Not concerned at all / A little concerned / Somewhat concerned / Moderately concerned / Very concerned / I prefer not to answer)

2. The impact of genetic variants with unknown significance on the current polygenic risk components.

(Not concerned at all / A little concerned / Somewhat concerned / Moderately concerned / Very concerned / I prefer not to answer)

3. The impact of yet to be identified genetic variants on the outcome of the polygenic risk component of the CVD risk score.

(Not concerned at all / A little concerned / Somewhat concerned / Moderately concerned / Very concerned / I prefer not to answer)

4. The variability of the CVD risk score prediction across populations.

(Not concerned at all / A little concerned / Somewhat concerned / Moderately concerned / Very concerned / I prefer not to answer)

5. The score providing a risk estimate is calculated at a single time point.

(Not concerned at all / A little concerned / Somewhat concerned / Moderately concerned / Very concerned / I prefer not to answer)

6. The possibility of risk estimates changing with age.

(Not concerned at all / A little concerned / Somewhat concerned / Moderately concerned / Very concerned / I prefer not to answer)

7. The potential adverse impacts on life or other insurance policies for patients receiving CVD risk information.

(Not concerned at all / A little concerned / Somewhat concerned / Moderately concerned / Very concerned / I prefer not to answer)

8. The test results' usefulness in disease management and prevention.

(Not concerned at all / A little concerned / Somewhat concerned / Moderately concerned / Very concerned / I prefer not to answer)

9. Do you believe there are additional concerns about the risk score that have not been mentioned above?

(Yes / No / I prefer not to answer)

If yes, please elaborate.

BENEFITS

In relation to the precision medicine-based CVD risk stratification tool proposed in the video, please select the corresponding box that BEST fits the level of benefit with each statement:

1. Access to CVD screening approaches tailored to African populations.

(Not beneficial at all / A little beneficial / Somewhat beneficial / Moderately beneficial / Very beneficial / I prefer not to answer)

2. The provision of population-specific CVD risk estimates.

(Not beneficial at all / A little beneficial / Somewhat beneficial / Moderately beneficial / Very beneficial / I prefer not to answer)

3. The provision of genetic and environmental CVD risk information to clinicians.

(Not beneficial at all / A little beneficial / Somewhat beneficial / Moderately beneficial / Very beneficial / I prefer not to answer)

4. The provision of genetic and environmental CVD risk information to patients.

(Not beneficial at all / A little beneficial / Somewhat beneficial / Moderately beneficial / Very beneficial / I prefer not to answer)

5. The provision of information for family planning.

(Not beneficial at all / A little beneficial / Somewhat beneficial / Moderately beneficial / Very beneficial / I prefer not to answer)

6. The provision of information for other family members.

(Not beneficial at all / A little beneficial / Somewhat beneficial / Moderately beneficial / Very beneficial / I prefer not to answer)

7. Adaption of prevention and treatment strategies based on a patient's CVD risk score.

(Not beneficial at all / A little beneficial / Somewhat beneficial / Moderately beneficial / Very beneficial / I prefer not to answer)

8. Greater incentive for patients to adopt prevention options including lifestyle changes.

(Not beneficial at all / A little beneficial / Somewhat beneficial / Moderately beneficial / Very beneficial / I prefer not to answer)

9. Do you believe there are additional benefits about the risk score that have not been mentioned above?

(No / Yes / I prefer not to answer)

If yes, please elaborate

10. In relation to the precision medicine-based CVD risk Strongly disagree stratification tool proposed, do you believe the Disagree expected health benefits outweigh the expected negative consequences to justify undertaking the risk assessment?

(Strongly disagree / Disagree / Neutral / agree / Strongly agree / I prefer not to answer)

CONFIDENCE IN APPLYING A PRECISION MEDICINE-BASED CVD RISK STRATIFICATION APPROACH
If the precision medicine-based CVD risk stratification tool proposed in the video was available, please select the corresponding box that BEST fits your level of confidence with each statement:

1. USING the CVD risk score for CVD screening.

(Not confident at all / A little confident / Somewhat confident / Moderately confident / Very confident / I prefer not to answer)

2. SUGGESTING the CVD risk score assessment for a patient.

(Not confident at all / A little confident / Somewhat confident / Moderately confident / Very confident / I prefer not to answer)

3. INTERPRETING the results obtained from the CVD risk score test.

(Not confident at all / A little confident / Somewhat confident / Moderately confident / Very confident / I prefer not to answer)

4. SHARING a patient's CVD risk score test results with them.

(Not confident at all / A little confident / Somewhat confident / Moderately confident / Very confident / I prefer not to answer)

5. EXPLAINING a PATIENT'S RISK based on their CVD risk score test results.

(Not confident at all / A little confident / Somewhat confident / Moderately confident / Very confident / I prefer not to answer)

6. EXPLAINING the potential ADVERSE IMPACTS on life or other insurance policies for patients receiving CVD risk information.

(Not confident at all / A little confident / Somewhat confident / Moderately confident / Very confident / I prefer not to answer)

7. If the precision medicine-based CVD risk stratification tool proposed was available, what is A little comfortable your level of comfort with adapting your PREVENTION STRATEGIES on the patient's CVD risk score results?

(Not comfortable at all / Somewhat comfortable / Moderately comfortable / Very comfortable / I prefer not to answer)

8. If the precision medicine-based CVD risk stratification tool proposed was available, what is your level of comfort with adapting your TREATMENT STRATEGIES based on the patient's CVD risk score results?

(Not comfortable at all / Somewhat comfortable / Moderately comfortable / Very comfortable / I prefer not to answer)

EXPECTATIONS IN APPLYING A PRECISION MEDICINE-BASED CVD RISK STRATIFICATION APPROACH
If the precision medicine-based CVD risk score is implemented on a population level to guide CVD prevention and screening programs, please select the corresponding box that BEST fits with the role clinicians will have?

1. CHOOSING the test for your patient

(No role / Supporting role / Primary role / Unsure / I prefer not to answer)

2. REFERRING the patient to a genetic specialist or counsellor

(No role / Supporting role / Primary role / Unsure / I prefer not to answer)

3. EXPLAINING the test to patients

(No role / Supporting role / Primary role / Unsure / I prefer not to answer)

4. INTERPRETING test results
(No role / Supporting role / Primary role / Unsure / I prefer not to answer)

5. SHARING results with patients
(No role / Supporting role / Primary role / Unsure / I prefer not to answer)

6. GUIDING treatment approach
(No role / Supporting role / Primary role / Unsure / I prefer not to answer)

7. If available, what is the likelihood that the precision medicine-based CVD risk score will influence your patient care in the future?
(No difference / Unlikely / Very unlikely Likely / Very likely / I prefer not to answer)

8. If the precision medicine-based CVD risk score is developed, when do you believe such a program could be available in South Africa's public clinical setting?
(Less than 1 year / 1 to 2 years / 2 to 5 years / 6 to 10 years / More than 10 years / Not implemented / Other / I prefer not to answer)

If other, please elaborate.

9. If the precision medicine-based CVD risk score is implemented nationally to guide prevention and screening programs, who do you believe will need to fund the testing?
National/provincial government / Private medical scheme / Patients / All of the above / Other / I prefer not to answer)

If other, please elaborate

BARRIERS TO THE IMPLEMENTATION OF A PRECISION MEDICINE-BASED CVD RISK STRATIFICATION APPROACH IN PUBLIC PRACTICE
Please select the corresponding box that BEST fits the level of impact each item may have on the ability to incorporate the precision medicine-based CVD risk stratification score into public practice.

1. Shortage of healthcare personnel
(No impact / Little impact / Some impact / Moderate impact / Strong impact / I prefer not to answer)

2. Shortage of genetic service capacity (specialists, counsellors, scientists, etc.)
(No impact / Little impact / Some impact / Moderate impact / Strong impact / I prefer not to answer)

3. Limited access to genetic services
(No impact / Little impact / Some impact / Moderate impact / Strong impact / I prefer not to answer)

4. Limited awareness of precision medicine
(No impact / Little impact / Some impact / Moderate impact / Strong impact / I prefer not to answer)

5. Lack of precision medicine training or education
(No impact / Little impact / Some impact / Moderate impact / Strong impact / I prefer not to answer)

6. Lack of clinical guidelines on precision-medicine based practice
(No impact / Little impact / Some impact / Moderate impact / Strong impact / I prefer not to answer)

7. Competing health interests
(No impact / Little impact / Some impact / Moderate impact / Strong impact / I prefer not to answer)

8. Physician time constraints
(No impact / Little impact / Some impact / Moderate impact / Strong impact / I prefer not to answer)

9. Time required for patient education
(No impact / Little impact / Some impact / Moderate impact / Strong impact / I prefer not to answer)

10. Cost of genomic testing
(No impact / Little impact / Some impact / Moderate impact / Strong impact / I prefer not to answer)

11. Lack of a willing funder

(No impact / Little impact / Some impact / Moderate impact / Strong impact / I prefer not to answer)

12. Questions about insurance coverage of the testing

(No impact / Little impact / Some impact / Moderate impact / Strong impact / I prefer not to answer)

13. Patient's concerns about moral/ethical implications

(No impact / Little impact / Some impact / Moderate impact / Strong impact / I prefer not to answer)

14. Cultural perceptions of genomic testing

(No impact / Little impact / Some impact / Moderate impact / Strong impact / I prefer not to answer)

15. Do you believe there are additional barriers to incorporating the score than those not mentioned above?

(No / Yes / I prefer not to answer)

If other, please specify.

Table S2: Multivariate analysis of respondents' mean (SD) knowledge, confidence, and perception scores (n =104)

Factor	Effect group	Reference group	Model coefficients *(95% CI)*	Standard error	Standardized coefficients (β)	*p* value
**Mean knowledge score** *(F-stat: 6.555, p-value: 8.715 x 10^{-7}, adj R-squared: 0.3014)*						
**Medical group**	Trainee	Clinician	0.849 *(0.15 – 0.84)*	0.176	0.326	*5.75 x 10^{-3}****
**Gender**	Male	Female	0.083 *(-0.14 - 0.30)*	0.111	0.062	*0.459*
**Clinical experience**	≥ 8 years	< 8 years	0.021 *(-0.25 - 0.29)*	0.136	0.016	*0.879*
**Postgraduate qualification**	Yes	No	0.242 *(-0.02 - 0.50)*	0.130	0.183	*0.065*
**Medical research involvement**	Yes	No	0.290 *(0.03- 0.55)*	0.132	0.223	*0.030 **
**CVD screening**	Yes<br>Sometimes	No	0.203 *(0.05 - 0.45)*<br>0.074 *(-0.20 - 0.35)*	0.127<br>0.137	0.152<br>0.052	*0.114*<br>*0.592*
**Genetics training**	Yes	No	0.758 *(0.46- 1.06)*	0.151	0.434	*2.48 x 10^{-6} ****
**Mean perception score** *(F-stat: 2.962, p-value: 5.391 x 10^{-3}, adj R-squared: 0.1357)*						
**Medical group**	Trainee	Clinician	0.283 (-0.28 – 0.84)	0.281	0.133	*0.317*
**Gender**	Male	Female	-0.216 (-0.57 – 0.14)	0.179	-0.115	*0.227*
**Clinical experience**	≥ 8 years	< 8 years	0.088 (-0.36 - 0.53)	0.224	0.048	*0.694*
**Postgraduate qualification**	Yes	No	0.020 (-0.40 - 0.44)	0.209	0.011	*0.923*
**Medical research involvement**	Yes	No	0.371 (-0.05 -0.79)	0.213	0.201	*0.085*
**CVD screening**	Yes<br>Sometimes	No	0. 746 (0.34 - 1.15)<br>0.533 (-0.09 -0.97)	0.205<br>0.222	0.396<br>0.263	*4.49 x 10^{-4} ****<br>*0.018 **
**Genetics training**	Yes	No	0.349 (-0.14 - 0.84)	0.247	0.139	*0.161*
**Mean confidence score** *(F-stat: 2.797, p-value: 8. 941 x 10^{-3}, adj R-squared: 0.1279)*						
**Medical group**	Trainee	Clinician	0.658 (0.00 – 1.32)	0.331	0.271	*0.050 **
**Gender**	Male	Female	-0.094 (-0.31 -0.50)	0.183	0.044	*0.1932*
**Clinical experience**	≥ 8 years	< 8 years	0.034 (-0.54 -0.48)	0.257	-0.016	*0.8935*
**Postgraduate qualification**	Yes	No	-0.010 (0.050 -0.48)	0.247	-0.004	*0.969*
**Medical research involvement**	Yes	No	0.928 (0.43 -1.43)	0.252	0.443	*3.91 x 10^{-4} ****
**CVD screening**	Yes<br>Sometimes	No	0.635 (0.17 -1.10)<br>0.369 (-0.14 -0.88)	0.236<br>0.258	0.298<br>0.162	*8.46 x 10^{-3} ****<br>*0.155*
**Genetics training**	Yes	No	-0.058 (-0.61 -0.50)	0.279	-0.020	*0.837*

*CVD – cardiovascular diseases; p-value thresholds: '***' = p ≤ 0.001, '**' = p ≤ 0.01, '*' = p ≤ 0.05*

Table S3: Clinician's responses relating to their expectations of a PM-based CVD risk stratification tool in the South African public health setting

Variable	Variable options	N	%
**Expected role (N =104)**			
**Choose the CVD risk score**	Primary	59	56.7
	Supporting	37	35.6
	Unsure	5	4.8
	No role	2	1.9
	I prefer not to answer	1	1.0
**Refer a patient for the CVD score**	Primary	75	72.1
	Supporting	23	22.1
	Unsure	3	2.9
	No role	2	1.9
	I prefer not to answer	1	1.0
**Explain CVD score results**	Primary	47	45.2
	Supporting	51	49
	Unsure	3	2.9
	No role	2	1.9
	I prefer not to answer	1	1.0
**Interpret CVD score results**	Primary	46	44.2
	Supporting	45	43.3
	Unsure	5	4.8
	No role	7	6.7
	I prefer not to answer	1	1
**Share CVD score results**	Primary	64	61.5
	Supporting	35	33.7
	Unsure	2	1.9
	No role	4	3.8
	I prefer not to answer	1	1.0
**Use the CVD score results to guide care approach**	Primary	72	69.2
	Supporting	24	23.1
	Unsure	3	2.9
	None	4	3.8
	I prefer not to answer	1	1.0
Likely behaviour shift (N = 100)			
**If available, likelihood CVD score will influence patient care**	Very likely	35	35.0
	Likely	54	54.0
	No difference	4	4.0
	Unlikely	4	4.0
	Very unlikely	3	3.0
Funder (N =100)			
**Funder**	National/provincial governments	36	36.0
	Private medical scheme	6	6.0
	Patients	4	4.0
	All of the above (Governments, Private medical schemes and patients)	52	52.0
	Other	2	2.0
Time horizon (N = 101)			
**Time horizon**	Less than 1 year	1	1.0
	1 to 2 years	4	4.0
	2 to 5 years	21	20.8
	6 to 10 years	39	38.6
	More than 10 years	36	25.6

CVD – cardiovascular diseases
